# Deacetylation of Histone H4 Accompanying Cardiomyogenesis is Weakened in HDAC1-Depleted ES Cells

**DOI:** 10.3390/ijms19082425

**Published:** 2018-08-16

**Authors:** Orazio Angelo Arcidiacono, Jana Krejčí, Jana Suchánková, Eva Bártová

**Affiliations:** 1 Institute of Biophysics of the Czech Academy of Sciences, Královopolská 135, Brno 612 65, Czech Republic; o.arcidiacono@gmail.com (O.A.A.); suchankova.jana@gmail.com (J.S.); 2 Faculty of Sciences, Masaryk University, Kamenice 753/5, Brno 625 00, Czech Republic

**Keywords:** cardiomyocytes, histones H3 and H4, embryonic stem cells, epigenetics, HDAC1

## Abstract

Cell differentiation into cardiomyocytes requires activation of differentiation-specific genes and epigenetic factors that contribute to these physiological processes. This study is focused on the in vitro differentiation of mouse embryonic stem cells (mESCs) induced into cardiomyocytes. The effects of clinically promising inhibitors of histone deacetylases (HDACi) on mESC cardiomyogenesis and on explanted embryonic hearts were also analyzed. HDAC1 depletion caused early beating of cardiomyocytes compared with those of the wild-type (wt) counterpart. Moreover, the adherence of embryonic bodies (EBs) was reduced in HDAC1 double knockout (dn) mESCs. The most important finding was differentiation-specific H4 deacetylation observed during cardiomyocyte differentiation of wt mESCs, while H4 deacetylation was weakened in HDAC1-depleted cells induced to the cardiac pathway. Analysis of the effect of HDACi showed that Trichostatin A (TSA) is a strong hyperacetylating agent, especially in wt mESCs, but only SAHA reduced the size of the beating areas in EBs that originated from HDAC1 dn mESCs. Additionally, explanted embryonic hearts (e15) responded to treatment with HDACi: all of the tested HDACi (TSA, SAHA, VPA) increased the levels of H3K9ac, H4ac, H4K20ac, and pan-acetylated lysines in embryonic hearts. This observation shows that explanted tissue can be maintained in a hyperacetylation state several hours after excision, which appears to be useful information from the view of transplantation strategy and the maintenance of gene upregulation via acetylation in tissue intended for transplantation.

## 1. Introduction

Over the years, many techniques leading to cardiomyocyte differentiation and isolation have been established. Unfortunately, by the use of these differentiation protocols, beating colonies of cardiomyocytes induced in vitro were not physiologically identical to cardiomyocytes isolated in vivo. Additionally, there have been several attempts to generate permanent, early cardiac cell lines. For example, H9c2-derived cells from embryonic rat heart [1], embryonal avian heart [2], transgenic mice with myocardial tumors [3], or neonatal rat myocardial cells transfected with SV-40 large T antigen [4] have been tested. In these cases, the established cell lines were not effective in studies of physiological processes leading to functional cardiomyocytes. The main reason for this result was that these cell lines were able to grow in culture for only a few passages because optimal cultivation conditions, including the composition of the cell culture media, for their growth in vitro had not been fully established [5]. From this view, Wobus et al. [6] studied the effect of retinoic acid on cardiomyocyte differentiation; these authors specifically analyzed cell induction into ventricle-like cardiomyocytes. In their protocol, the authors induced cardiomyocytes with Purkinje- and ventricle-like markers while a reduced number of pacemaker- and atrium-like cells were observed.

Cardiomyocyte differentiation has also been effectively studied in mouse and human embryonic stem cells (mESCs). These cells are characterized by pluripotency, self-renewal, broad differentiation plasticity, a relatively stable karyotype, and the ability to differentiate into cells from all three germ layers (ectoderm, mesoderm, and endoderm). Embryonic stem cells can be isolated from the inner cell mass (ICM) of blastocysts, and these cells are considered to be pluripotent. In this regard, Wobus et al. [7] explained that in mice, the fertilized oocyte and blastomeres of two-, four-, and eight-cell-stage embryos are totipotent, while cells from the ICM and the embryonic ectoderm and the primordial germ cells from fetal stages are only pluripotent. When transferred into an early embryo, these cells can physiologically mimic all cells of the embryo but not the placental tissue, and, therefore, these cells are not able to generate an organism.

For experimental studies, it is very useful to analyze the differentiation potential of three-dimensional embryoid bodies (EBs) generated from ESCs in “hanging drops”. EB development shows a multicellular arrangement like those of skeletal [8] cells, neuronal cells [9], blood vessels [10], and epithelial cells [11]. Moreover, EBs consist of various extracellular matrix components (collagen, laminin, nidogen, and fibronectin) [12,13]. In this complex multicellular structure, spontaneous beating cardiomyocytes appear between the epithelial layer and basal layer of mesenchymal cells [14]. In these highly specialized cells, it is generally accepted that beating attributes, such as beating frequency and an appearance of cardiomyocytes markers, including α-actinin, represent a tool for how to mimic cardiomyogenesis in vitro [15,16].

Cardiomyocytes are also characterized by specific epigenetic features. Epigenetics refers to heritable modifications of DNA, RNA, and histones. Epigenetic regulation, playing a role not only in cardiomyogenesis, includes DNA methylation and the function of non-coding RNAs or histone post-translational modifications (PTMs). Histone modifications are, for example, regulated by enzymes, such as kinases, histone acetyltransferases (HATs), histone deacetylases (HDACs), or histone methyltransferases/demethylases [17]. These epigenetic features can be affected by many factors, including pollution in the environment, diet (referred to as epi-diet), and disease progression or epigenetic therapy. In cardiomyocytes, for example, the p300 transcriptional co-activator, which is considered to be a HAT (responsible for GATA-4 acetylation) potentiates GATA-4′s DNA binding property [18]. Moreover, HDAC inhibitors (HDACi), including TSA, promote the differentiation of ESCs into cardiomyocytes; thus, it appears to be evident that HDACs in general play a role in cardiomyogenesis. As an example, a unique study of human hearts showed that HDAC4 inhibits the expression of pro-hypertrophic genes by recruiting a histone methyltransferase, SUV39H1, to regulatory elements for these loci that become methylated on histone H3 lysine-9 (H3K9) [19]. HDAC4 associates not only with SUV39H1 but also with the DNA binding transcription factor MEF2 (myocyte enhancer factor 2) and heterochromatin protein 1 (HP1). This nuclear event is essential for HDAC4-mediated downregulation of pro-hypertrophic genes. Conversely, from the view of hypertrophic gene activation, HDAC4 was found to be exported from the nucleus, and thus, the MEF2 factor was released to interact with histone acetyltransferases (HATs) that contribute to the upregulation of these genes. Cardiovascular diseases (CVD), including coronary artery diseases (CAD), such as angina and myocardial infarction, and other diseases, such as stroke, heart failure, rheumatic heart disease, hypertensive heart, and others, are one of the major reasons for death in the world. Unfortunately, the incidence has dangerously increased over the past 100 years. CVD is a group of multifactorial disorders linked to genetic risk factors [20]. These factors (mostly primary diseases) include hypertension, diabetes mellitus, familial hypercholesterolaemia, and familial hyperlipidaemia; all these diseases are characterized by a specific genetic background [21]. Thus, studies with experimental animals, focused not only on the physiology but also on the pathophysiology of cardiac tissue, enable a better understanding of the molecular mechanisms of CVD. From this view, it is well-known that epigenetic regulation plays a fundamental regulatory role on genes in which their expression is associated with CVD risk. Thus, an understanding of epigenetic events associated with CVD is essential from the view of potential therapy using epi-drugs. In this scenario, epigenetic approaches, especially the clinical application of HAT or HDAC inhibitors, are promising for new therapeutic strategies [22]. For example, an understanding of how histone acetylation affects cardiomyogenesis and how HATs and HDACs work during this process could initiate new and promising therapeutic approaches. The connection between changes in histone acetylation and cardiac hypertrophy was demonstrated by Gusterson and colleagues. These authors showed that overexpression of the transcriptional co-activators CREB-binding protein (CBP) and p300 HAT can induce cardiac hypertrophy due to the HAT activity of these proteins [23]. Antos et al. demonstrated that inhibition of HDACs by TSA shut down cardiac hypertrophy without affecting cell viability [24]. Cao et al. showed that autophagy processes in heart failure represent a target for therapy by HDACs inhibitors [25], and Liu et al. additionally documented the epigenetic background of the most common clinical cardiac arrhythmia: atrial fibrillation (AF). Mice overexpressing the HopX protein develop cardiac hypertrophy, which influences AF. Using a specific HDAC inhibitor (TSA), researchers have reduced atrial arrhythmogenesis as well as reversed atrial fibrosis [26]. Montogomery et al. highlighted the role of HDAC1 and HDAC2 in the control of cardiac function. Deletion of both genes (Hdac1 and Hdac2) in a mouse model resulted in early death caused by fetal arrhythmia or dilated cardiomyopathy [27]. Moreover, Montomery et al. showed that the phenotype of cardiac-specific Hdac3 gene deletion is distinct from those mutations in other Hdac genes [28]. This observation implies that the role of the Hdac3 gene is important for cardiac function. From this view, McKinsey, using a rodent model for heart failure, obtained promising data about the therapeutic potential of HDAC inhibitors [29]. According to the above-mentioned data, it appears likely that HDAC function, modified by HDAC inhibitors, is important for cardiovascular therapeutic applications. Thus, here we address the question of how HDAC1 depletion affects mESC differentiation into cardiomyocytes and how the histone signature, especially histone H3 and H4 acetylation or H3 methylation, is affected by HDACi treatment of HDAC1 wild-type (wt) and HDAC1 double knockout (dn) mESCs. Additionally, we analyzed embryonic heart epigenetic features. We tested clinically promising HDACi, including TSA (trichostatin A), SAHA (suberoylanilide hydroxamic acid, syn. vorinostat), and VPA (valproic Acid), on explanted mouse hearts. Our main hypothesis was that HDACi would modulate the epigenome of cardiac cells and HDAC1 depletion would affect the efficiency of cardiac differentiation.

## 2. Results

### 2.1. The Beating of Cardiomyocytes Studied in wt and HDAC1 dn Cells

**Figure 1:**
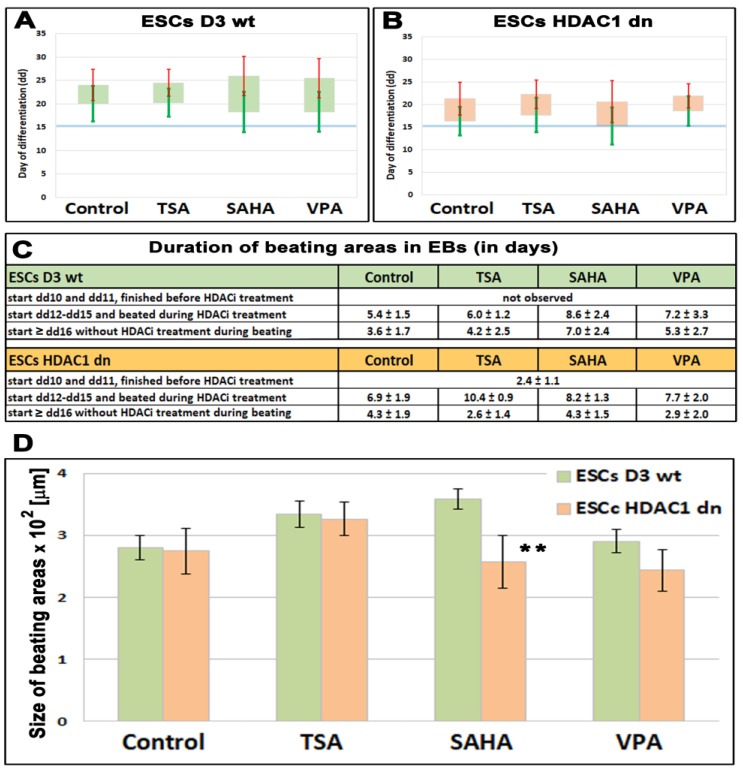
The beating of cardiomyocytes in differentiated HDAC1 wild-type (wt) and HDAC1 double knockout (dn) mouse embryonic stem cells (ESCs). Colonies of ESCs (embryoid bodies, EBs) were monitored every day in (**A**) HDAC1 wt mESCs, and (**B**) HDAC1 dn mESCs. Bars in panels (**A**,**B**) show an average day of cardiomyocytes’ beating. Green standard errors (scales) show a start of beating and red standard errors (scales) show the end of beating (scale of the *y*-axis is in days). The observation was performed in non-treated wt and HDAC1 dn mESCs induced to cardiac differentiation and these cells treated by HDACi (TSA, SAHA, and VPA). (**C**) A duration of beating areas in EBs of non-treated and EBs treated by TSA, SAHA, and VPA is shown. (**D**) A size of beating areas in embryonic bodies is documented. Asterisks (**) show a statistically significant difference at *p* ≤ 0.05.

Cardiomyocyte differentiation was induced in the EBs of wt and HDAC1-depleted ESCs, and the beating was monitored every day. Cardiomyocytes beat from day 12 to day 25, with an average differentiation on the 20th day, but the beating period was affected by HDAC1 depletion (Figure 1A,B). Briefly, the beating period was not identical in the non-treated wt and the non-treated HDAC1 dn cells. HDAC1 wt mESCs started to beat at day 12–15 after differentiation, and beating lasted approximately 5 days (Figure 1C). However, HDAC1 dn mESCs started to beat on the 10th day after differentiation, and when these cells began to beat in this early interval, the period of beating was 2.5 days (Figure 1C). HDAC inhibitors, including TSA, SAHA, and VPA, had an ability to prolong the time of the beating irrespective of HDCA1 depletion (Figure 1C). The beating time was 6–7 days in the case of HDCAi treatment (Figure 1C). The size of the beating area was only reduced in the SAHA-treated HDAC1 dn cells (Figure 1D). These data show that HDAC inhibitors, especially SAHA, have an effect on the beating of cardiomyocytes that were differentiated from mESCs.

### 2.2. Adherence of Embryonic Bodies Is Affected by HDAC1 Depletion

**Figure 2:**
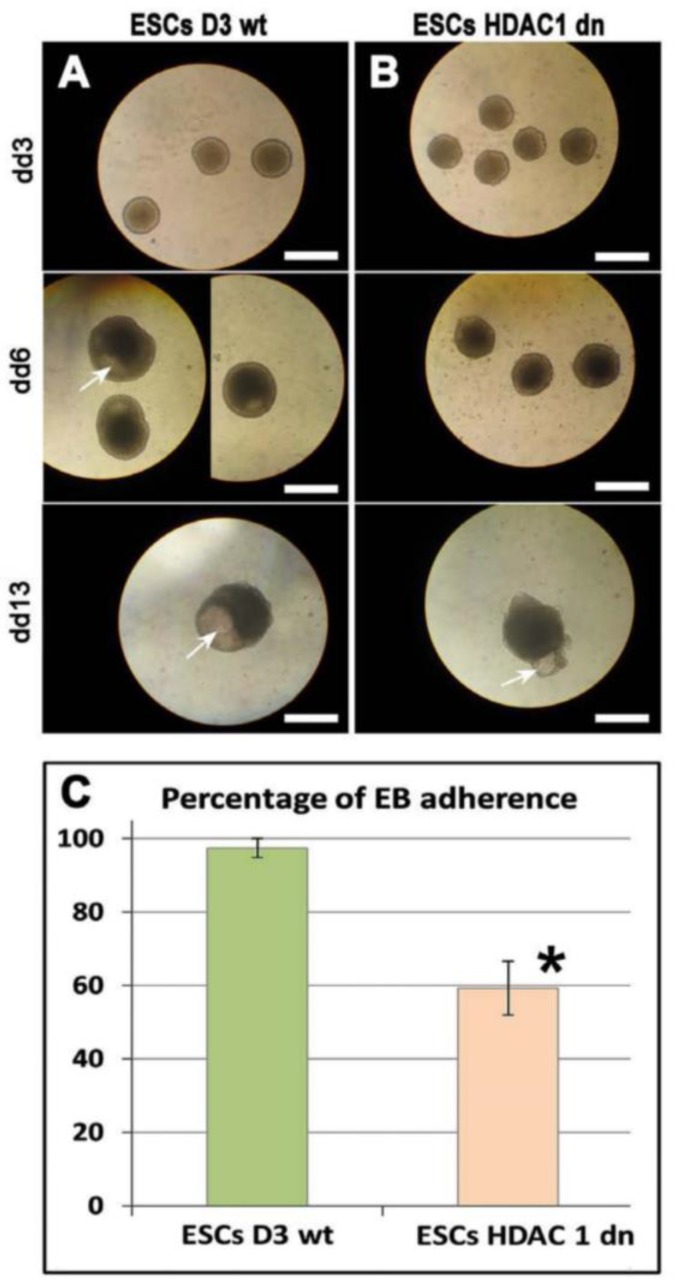
Studies on EB formation and adherence in HDAC1 wt and HDAC1 dn mESCs. Under transmitted light microscopy and through the use of bright-field microscopy, the formation of EBs was inspected at day 3 (dd3), 6 (dd6), and 13 (dd13) in (**A**) HDAC1 wt mESCs and (**B**) HDAC1 dn mESCs. Cavity formation in EBs is shown by white arrows. Scale bars show 500 µm. Adherence of EBs was reduced in HDAC1 dn mESCs as shown in panel (**C**). The asterisk shows a statistically significant difference at *p* ≤ 0.05 (*).

Embryonic bodies (EBs) represent an important cellular model for the study of embryonic development and are a useful tool for testing the role of pluripotency in vitro and the induction of the differentiation processes. The use of EBs is highly respected only when the cells have an ability to fully differentiate within these three-dimensional structures. In EBs, we observed the formation of the cavity. The cavitation should mimic the formation of the developing body cavity. The cavitation can represent a phenomenon linked to not only pericardial formation but also to the lateral plate mesoderm cavities, such as the pleural and peritoneal cavities. Interestingly, in EBs generated from wt mESCs, the cavity appears early, at day 6 (dd6) of differentiation. In HDAC1 dn mESCs, the appearance of cavitation started later than in wt cells (dd10), and here we show day 13 (dd13) that was additionally characterized by cavity malformations (Figure 2A).

Here, we also study the adherence of EBs using wide-field microscopy, and the EBs were monitored on day dd3, dd6, and dd13 of cell cultivation and differentiation. The observations were performed using transmitted light microscopy (Figure 2A,B). We calculated the percentage of adherent EBs as shown in Figure 2C. In comparison to wt cells, we found a reduced number of adherent EBs that were generated from the HDAC1 dn mESCs. This result was statistically significant at *p* ≤ 0.05. 

### 2.3. Differentiation-Specific Deacetylation of Histone H4 was Weakened in HDAC1-Depleted Cells

**Figure 3:**
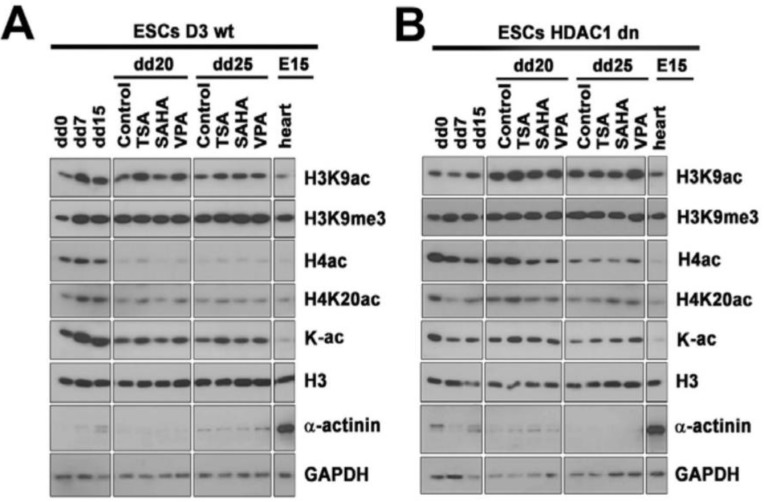

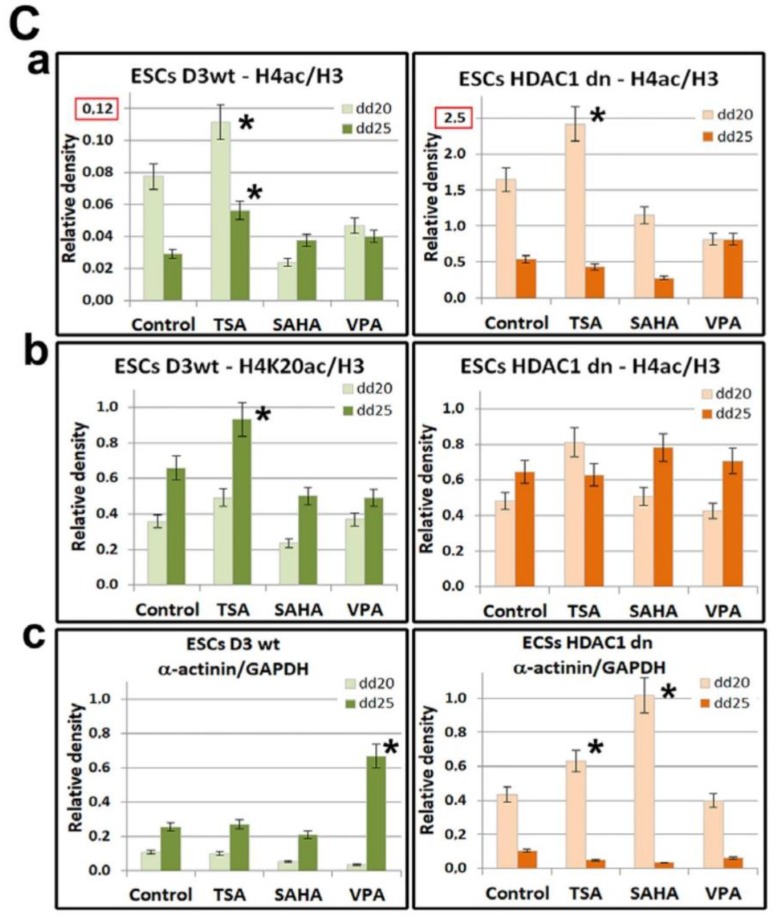
Histone acetylation and methylation in HDAC1 wt and HDAC1 dn mESCs induced into cardiomyocytes and treated with HDACi. The level of H3K9ac, H3K9me3, H4ac, H4K20ac, pan-acetylated lysines (K-ac), and α-actinin in (**A**) HDAC1 wt mESCs and (**B**) HDAC1 dn mESCs. In three biological replicates, Western blots were performed on one gel. For the data presented in panel A or B, the gel was separated by Photoshop to show samples that were compared in one relevant subset. Data on histone levels were normalized to the level of histone H3 and non-histone proteins were normalized and quantified to the level of GAPDH (**C**). In wt and HDAC1 dn non-treated cells and in TSA-, SAHA-, or VPA-treated mESCs, panel (**Ca**) shows the levels of H4ac, (**Cb**) shows H4K20ac, and (**Cc**) shows the levels of α-actinin. The total protein levels were measured using a µQuant spectrophotometer for each sample, and an identical protein amount was loaded on the gels. In panel (**A**,**B**), the levels of histone markers are also shown for embryonic hearts (e15). Quantification of the protein levels in panel (**C**) was performed using ImageJ software (NIH, freeware). Statistical analyses were performed using Student’s *t*-test; asterisks (*) in panel (**Ca**–**c**) show statistically significant differences at *p* ≤ 0.05. Note that the *y*-axis-scale in panel (**Ca**) is different (red frames) for the wt and HDAC1 dn cells for technical purposes. In panel (**Ca**), the level of H4ac is significantly less in the wt mESCs when compared with the HDAC1 dn cells (**Cb**).

Through the use of Western blots, we studied the level of selected histone markers in non-differentiated wt and HDAC1 dn mESCs or these cells differentiated into cardiomyocytes. Terminally differentiated cardiomyocytes were additionally treated by HDAC inhibitors, including TSA, SAHA, or VPA. In wt cells, we observed the deacetylation of histone H4 that accompany cardiomyocyte differentiation. Interestingly, H4 deacetylation was weakened in the HDAC1-depleted cells (Figure 3(A,B,Ca,Cb)).

We additionally studied H4K20ac, H3K9ac, pan-lysine acetylation, and H3K9me3 in wt and HDAC1 dn cells. In these histone markers, we observed a decrease in H4K20ac in differentiated wt cells and HDAC1 dn cells. H3K9ac increased significantly in differentiated HDAC1 dn cells (Figure 3A,B). The H3K9me3 level increased on day 7 (dd7) of differentiation and then maintained a relatively stable level in both the wt and HDAC1 mESCs induced to cardiac differentiation (Figure 3A,B).

Here, we also show the results of the effect of HDACi on levels of H4ac, H4K20ac, and α-actinin (Figure 3(Ca–c)). We observed that, as opposed to SAHA and VPA, trichostatin A induced H4 and H4K20 hyperacetylation in wt cells on day 20 or 25 of cardiac differentiation, and in HDAC1 dn cells, this was observed on the 20th day of differentiation (Figure 3(Ca,b)). We found a significant increase in wt mESCs treated by TSA and when observed on day 25 (dd25) of differentiation (Figure 3(Cb)). Moreover, the α-actinin level, which is a marker of cardiomyogenesis, was elevated in the VPA-treated and differentiated wt cells (dd25), and the increased level of α-actinin was significant in the HDAC1 dn cells treated by TSA or SAHA on the 20th day (dd20) of cardiac differentiation (Figure 3(Cc)).

**Figure 4:**
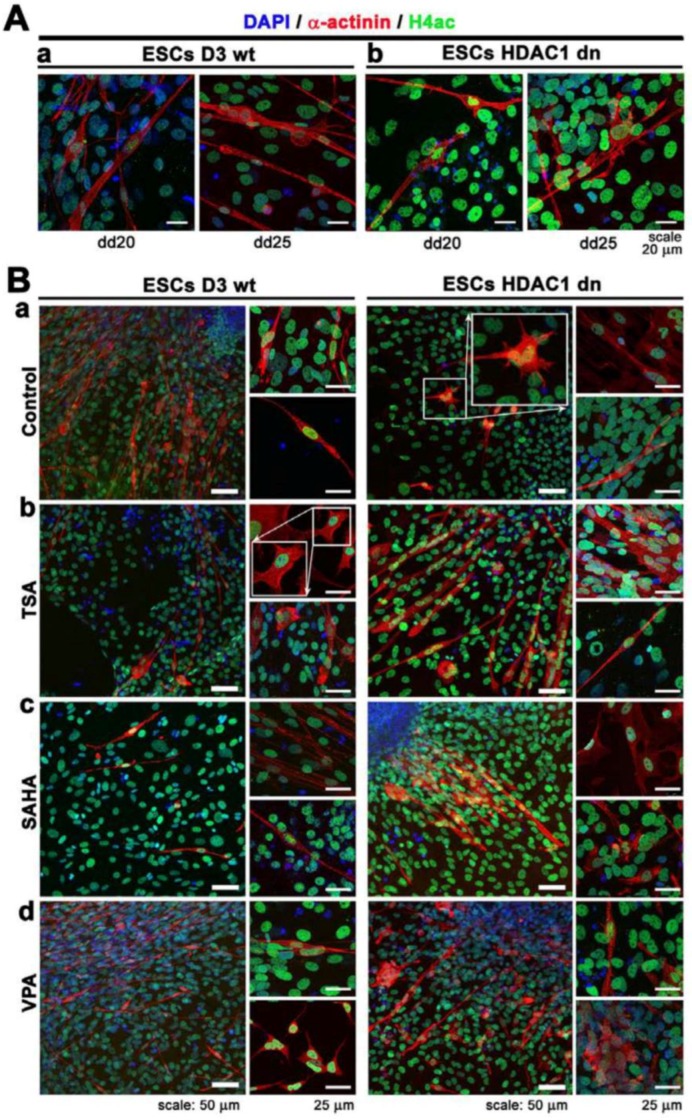
Distribution pattern and levels of histone H4 acetylation (green) and α-actinin (red) in (**Aa**) wt mESCs and (**Ab**) HDAC1 dn mESCs differentiated at dd20 and dd25. Panel (**Ba**) shows non-treated wt mESCs and HDCA1 dn mESCs at dd25, and panels (**Bb**–**d**) show wt mESCs and HDCA1 dn mESCs treated by HDACi at dd25: (**Bb**) TSA treatment, (**Bc**) SAHA treatment, and (**Bd**) VPA treatment. DAPI (blue) was used as a counterstain of the cell nuclei.

The differentiation-specific deacetylation effect, which was observed by Western blots (Figure 3A,B), was also confirmed by immunofluorescence combined with confocal microscopy (Figure 4A,B). From a cell morphology point of view, we observed that HDAC1-depleted cells were characterized by star-shaped α-actinin filaments (Figure 4(Ba), frame). Interestingly, the distribution pattern of the α-actinin filaments of the TSA-treated wt mESCs resembled tile-like contours (Figure 4(Bb)). This distribution pattern was observed in 70–80% of the α-actinin-positive wt cells. A similar distribution pattern was also observed in 40–50% of the SAHA-treated HDAC1 dn cells (Figure 4(Bc)). VPA changes only 5% of the wt cells to having a star-like shape (Figure 4(Bd), right bottom part in wt cells).

### 2.4. Histone Hyperacetylation Can Be Induced by HDACs Inhibitors in Hearts Explanted from Mouse Embryos at Stage e15

**Figure 5:**
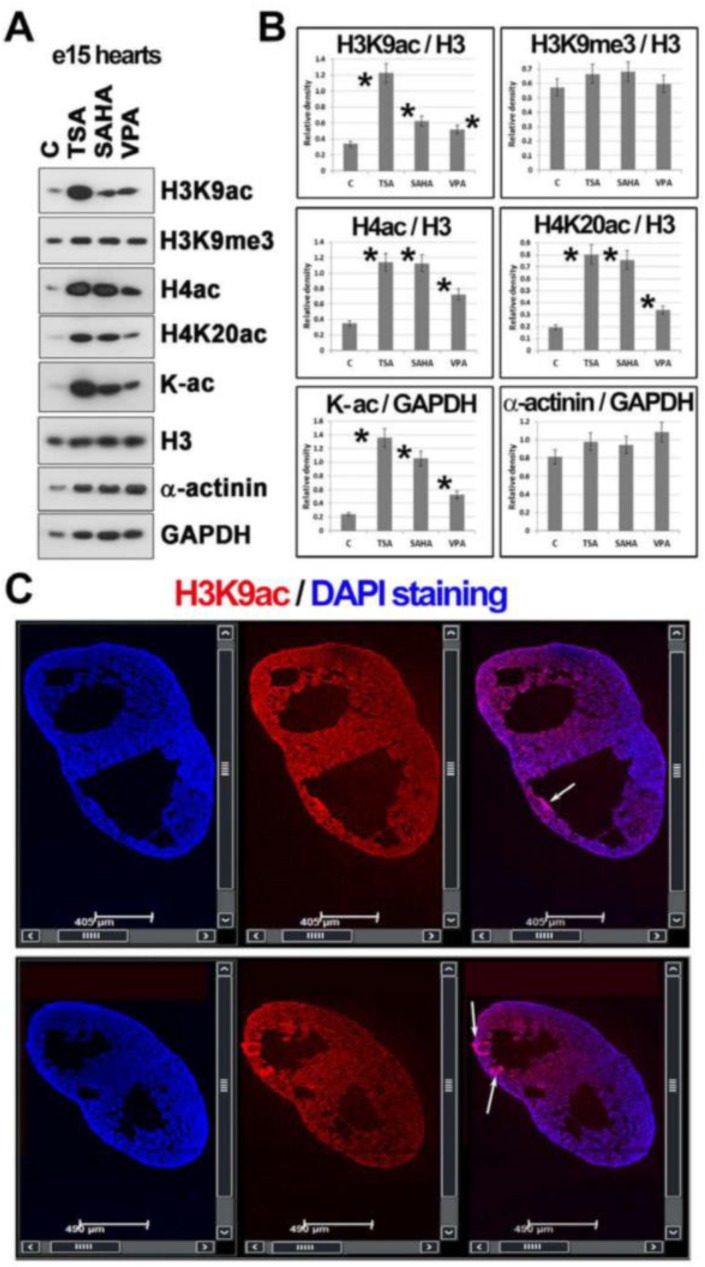
Histone post-translational modifications studied in mouse embryonic hearts (e15) treated with HDACi. (**A**) Western blots showed changes in H3K9ac, H3K9me3, H4ac, H4K20ac, pan-acetylated lysines (K-ac), and α-actinin in e15 embryonic hearts treated with HDACi (TSA, SAHA, and VPA). Data on histone levels were normalized to the level of histone H3 and non-histone proteins were normalized to the level of GAPDH. An identical protein amount for each experimental event was loaded on the gel. (**B**) Data from panel (**A**) were normalized to the relevant reference protein GAPDH, and the density of Western blot fragments was statistically analyzed using Student’s *t*-test; asterisks show statistically significant differences at *p* ≤ 0.05. GAPDH was used for data normalization, and α-actinin was used as a marker of cardiomyocytes. (**C**) The distribution pattern of H3K9ac (red) in the e15 mouse embryonic hearts is shown. DAPI (blue) was used as a counterstain of the cell nuclei. Arrows show the accumulation of H3K9ac in ventricular portions.

Explanted embryonic hearts (e15) also undergo histone hyperacetylation when treated with HDACi. We found that all tested HDACi (TSA, SAHA, VPA) increased the level of H3K9ac, H4ac, H4K20ac, and pan-acetylated lysines. TSA and SAHA were very strong hyperacetylating agents in terms of the total H4ac. When we focused on H4K20ac, the level of this epigenetic marker was also increased in e15 hearts (Figure 5A,B). However, pronounced H3K9ac was observed only after the treatment using TSA (Figure 5A,B). Using immunofluorescence and confocal microscopy, we additionally observed that H3K9ac is homogeneously distributed throughout e15 embryonic hearts, and this is additionally characterized by a nodal accumulation of H3K9ac in ventricular portions (Figure 5C).

Additionally, we studied the level of α-actinin as a marker of cardiomyocytes, but the level of this marker (when normalized to the level of GAPDH) was not affected by HDACi treatment of explanted hearts (Figure 5A,B). These data unambiguously show that even in explanted hearts, histones can be epigenetically modified using epi-drugs, which could be an important observation from the viewpoint of transplantation strategies.

## 3. Discussion

It is well-known that the differentiation of ESCs into cardiomyocytes is regulated by the specific subset of genes that are furthermore regulated by epigenetic factors, including HATs and HDACs [30,31]. The present study shows that HDAC1 depletion, to some extent, prevents total H4 deacetylation, which is an epigenetic marker of cardiomyogenesis (Figure 3A,B). Conversely, H3K9ac was relatively stable in differentiated wt mESCs, while HDAC1 dn mESCs that were induced to cardiac differentiation were characterized by a pronounced increase in H3K9ac (Figure 3B). In parallel, the H3K9me3 level began to be elevated in both tested mES cell lines from dd7 of cardiac differentiation, and then the level of this histone mark remained stable. Interestingly, differentiated cardiomyocytes from both wt and HDAC1 dn cells were characterized by a lower level of α-actinin in comparison to e15 embryonic hearts (Figure 3A,B). In detail, in wt mESCs, α-actinin was detectable at dd25 of cardiac differentiation, while in differentiated HDAC1 dn mESCs, the Western blot fragment showing α-actinin was visible at dd20 of differentiation and was even visible in the cells treated by HDACi (Figure 3A,B). VPA treatment increased the level of α-actinin at dd25 in wt cells, but TSA and SAHA in HDAC1 dn cells elevated the α-actinin level earlier, at dd20 (Figure 3(Cc)).

Here, we document that HDAC1 depletion has an effect not only on the histone signature, especially H4ac, but also on beating areas in EB-containing cardiomyocytes (Figure 1A,B and Figure 3(A–Ca)). From this view, the class I HDACs appear to be very important for cardiac differentiation and heart development. For example, HDAC2 promotes cardiac hypertrophy [32], and cardiac-specific deletion of HDAC3 in mice leads to cardiac hypertrophy and excessive myocardial lipid accumulation [28]. Moreover, HDAC6 regulates a structural and functional remodeling of atrial myocytes [33]. Here, we showed that HDAC1 depletion affects the timing related to the appearance of the cardiac marker α-actinin during in-vitro-induced cardiomyogenesis. Interestingly, the level of α-actinin was potentiated by HDACi treatment, likely due to hyperacetylation. This conclusion is in an agreement with Kawamura et al. [30] showing that TSA increased the acetylation of not only histones but also non-histone proteins, including the zinc finger protein GATA-4. This epigenetic event was induced during differentiation of ESCs into cardiomyocytes. These data document that non-histone proteins can also be affected by HDACs inhibitors. Similarly, Glozak et al. [34] showed that HATs and HDACs affect the function of non-histone proteins, including transcription factors, and their interaction potential. Moreover, HDACi are also functional, not only in cell lines cultivated in vitro but also in explanted mouse hearts in which we found that HDACi increased H4ac, H4K20ac, H3K9ac, and the acetylation of lysines (K-pan-acetylation) (Figure 5A,B). This is a very interesting observation that shows that even explanted organs, at least 3 h after experimental surgery, underwent histone signature changes. Thus, from the viewpoint of tissue transplantation, HDACi might be considered as potential maintainers of tissue epigenome. Moreover, protein hyperacetylation could be beneficial from the viewpoint of enhanced expression of genes playing a role in graft acceptance and subsequent tissue regeneration. Thus, our claim fits well with Tao et al. [35] suggesting a post-transplantation role of HDACi. These authors showed that HDACi can affect regulatory T cells (Tregs), increase acetylation of Foxp3 protein, and cause chromatin remodeling. Thus, epigenetic regulation via epi-drugs could be taken into account from the viewpoint of therapeutic applications in transplantation medicine.

## 4. Materials and Methods

### 4.1. Mouse ESCs Cultivation and Differentiation

Epigenetic aspects and the function of HDACs were studied in mESCs: wild-type mESCs-D3 line (mESCs wt, wild-type) [36] and mESCs that were deficient in HDAC 1 (HDAC1 dn mESCs) [37]. Mouse ESCs were cultivated on 0.2% gelatin-coated Petri dishes (valid for wt-cells) or Matrigel (#354277, Thermo Fischer Scientific, Rockford, IL, USA)-coated plastic dishes (valid for HDAC1dn-cells). Mouse ESCs were grown in Dulbecco’s Modified Eagle Medium (DMEM, Sigma-Aldrich, Prague, CZ) supplemented with penicillin and streptomycin, 0.1 mM non-essential amino acids, 1 ng/mL mouse leukemia inhibitory factor (LIF), 100 μM mono-thioglycerol, and 15% fetal bovine serum (FBS). Differentiation protocols showing mESC stimulation into cardiomyocytes via EBs were described in Kudová et al. [38]. Cells were cultured at 37 °C in a humidified atmosphere containing 5% CO_2_.

Cardio differentiation was initiated by seeding 400 cells per 30 μL into ES culture media without the addition of LIF factor using the “hanging drop” method. On the 3rd day of differentiation (dd3), the increased embryonic bodies (EBs) were plated onto non-adhesive (bacteriological) plastic dishes; on dd6, EBs were transferred to gelatin-coated culture dishes with DMEM/F12 (1:1) (#11320-033, Gibco, Paisley, UK) supplemented with insulin, transferrin, and selenium (ITS-100x, #41400-045, Gibco) (DMEM/F12-ITS). The adhesion efficiency was evaluated on dd7 and dd8. The serum-free DMEM/F12-ITS culture medium was changed every two days. On dd15, the HDACi (100 nM TSA, 8 μM SAHA, and 5 mM VPA) were applied for 3 h. The aim of treating the HDAC1-depleted cells using HDACs inhibitors was to potentiate the hyperacetylating effect of HDAC 1 depletion. Differentiation and cell monitoring were terminated on dd25.

### 4.2. Experimental Animals

To study epigenetic features of embryonic hearts, mouse strain C57Bl6 was used. Mice were housed in a specific pathogen-free (SPF) animal facility at the Institute of Biophysics of the Czech Academy of Sciences at a constant temperature of 21 °C and 60% humidity under a 12 h/12 h light/dark cycle with access to food and water ad libitum. All experiments with mice were performed according to the Agreement of the Ethics Commission of the Czech Academy of Sciences (document No.: 48/2016). After breading, embryos were explanted from female animals 15 days post conception (e15) and embryonic hearts were treated using HDAC inhibitors (200 nM TSA, 16 µM SAHA, and 15 mM VPA). HDACi were dissolved in DMEM supplemented by 10% of FBS. Explanted hearts were treated for 3 h in DMEM with 10% FBS, and the hearts were maintained at 37 °C in a humidified atmosphere containing 5% CO_2_. The concentrations of the HDACi were optimized in Večeřa et al. [39].

### 4.3. Immunostaining

After cell fixation with 4% paraformaldehyde, the interphase nuclei were permeabilized with 0.2% Triton X100 for 8 min, were treated with 0.1% saponin (Sigma-Aldrich) for 12 min, and then washed twice in PBS for 15 min. A solution of 1% bovine serum albumin in PBS was used for blocking of non-specific binding of antibodies. The procedure was performed at room temperature (RT) for 1 h. After washing with PBS for 15 min, samples were incubated overnight at 4 °C with the monoclonal antibodies of interest: α-actinin (#A-7811, Sigma-Aldrich), H3K9ac (#06-942, Merc Millipore, MA, USA) and H4ac (#382160, Merc Millipore). The next day, the cells were washed twice in PBS for 5 min and incubated for 1 h with the appropriate secondary antibody conjugated with the fluorochrome of interest (#A11032, Alexa Fluor 594 anti-mouse IgG, Life Technologies Corporation, Eugene, OR, USA; #ab150077, Alexa Fluor 488 anti-rabbit IgG, Abcam, Cambridge, UK). Immuno-stained preparations were washed three times in PBS for 5 min, and DAPI (4′,6-diamidino-2-phenylindole dihydrochloride; #10236276001, Roche, Prague, CZ) was used for counterstaining the cell nuclei.

### 4.4. Western Blot Analysis

For Western blot analysis, cell or tissue samples were washed with PBS and lysed in sodium dodecyl sulfate (SDS) lysis buffer (50 × 10^3^ mol/L Tris-HCl, pH 7.5; 1% SDS; 10% glycerol). Western blots were performed following Krejčí et al. [40] and Krejčí et al. [41]. For this research, we used antibodies against the following proteins: α-actinin (#A-7811, Sigma-Aldrich), H3K9ac (#06-942, Merc Millipore), H3K9me3 (#ab8898, Abcam), H4ac (#382160, Merc Millipore), H4K20ac (#720087, Thermo Fisher Scientific), acetylated Lysin (K) (#ab21623, Abcam), histone H3 (#ab1791, Abcam), and GAPDH (#sc-365062, Santa Cruz Biotechnology, Dallas, TX, USA).

### 4.5. Confocal Microscopy

For analyses, a Leica TSC SP-5 X or SP-8 confocal microscope was used and was equipped with a white light laser (470–670 nm in 1 nm increments), an argon laser (488 nm), and UV lasers (355 nm and 405 nm). We used objectives with the following magnification: 20× HCX PL APO lambda blue (20.0× 0.7 IMM UV, Leica Microsystems, Mannhein, Germany) and an oil objective HCX PL APO 63× lambda blue with a numerical aperture (N.A. = 1.4). To prevent photo bleaching of fluorochromes, we used hybrid detectors (HyD) for time-lapse confocal microscopy; alternatively, fixed cells were monitored by photomultipliers (PMTs). The LEICA LAS AF software was used for data acquisition and analysis. The following microscope settings were used: 1024 × 1024 pixels, 400 Hz, and 8 × zoom [42].

### 4.6. The Tile-Scanning

Cryo-sections (7 µm) of whole embryonic hearts (at stage e15) were stained by an antibody against H3K9ac (#06-942, Merc Millipore) to visualize the distribution pattern of this histone marker. For image acquisition, we used the “tile-scanning” mode and the following objective: HCX PL APO lambda blue 20.0× 0.7 IMM UV (Leica Microsystems). Scanning was performed at a resolution of 512 × 512 pixels, and for image reconstruction, we used the auto-stitched tile-scanning mode involving the smooth-scanning mode with a slow/fine speed accuracy (set in the Leica LAS AF software connected to the Leica SP5 X microscope) [39].

### 4.7. Statistical Analysis, Image Analysis, and Image Processing

For statistical analyses, the following software was used: ImageJ, Sigma Plot 2000, and the Leica 3D software suite (LEICA LAS AF). Sigma Plot software was used in to analyze statistically significant differences using Student’s *t*-test. The Leica 3D software suite (LEICA LAS AF) was used to measure the volume of the beating areas in EBs.

## Abbreviations

H3	histone H3
H4	histone H4
me1	mono-methylated histones
me2	di-methylated histones
me3	tri-methylated histones
H3K9me1/me2/me3	histone H3 methylated on lysine 9 (mono-; di-; tri-methylation)
H3K9ac	histone H3 acetylated on lysine 9
HDAC	histone deacetylases; HDAC1: histone deacetylase 1
HDACi	histone deacetylase inhibitors
Kac	lysine acetylation
PTM	post-translational modification
